# Causes of chest pain in primary care – a systematic review and meta-analysis

**DOI:** 10.3325/cmj.2015.56.422

**Published:** 2015-10

**Authors:** Jörg Haasenritter, Tobias Biroga, Christian Keunecke, Annette Becker, Norbert Donner-Banzhoff, Katharina Dornieden, Rebekka Stadje, Annika Viniol, Stefan Bösner

**Affiliations:** Department of General Practice/Family Medicine, Philipps University of Marburg, Marburg, Germany

## Abstract

**Aim:**

To investigate the frequencies of different and relevant underlying etiologies of chest pain in general practice.

**Methods:**

We systematically searched PubMed and EMBASE. Two reviewers independently rated the eligibility of publications and assessed the risk of bias of included studies. We extracted data to calculate the relative frequencies of different underlying conditions and investigated the variation across studies using forest plots, I^2^, tau^2^, and prediction intervals. With respect to unexplained heterogeneity, we provided qualitative syntheses instead of pooled estimates.

**Results:**

We identified 11 eligible studies comprising about 6500 patients. The overall risk of bias was rated as low in 6 studies comprising about 3900 patients. The relative frequencies of different conditions as the underlying etiologies of chest pain reported by these studies ranged from 24.5 to 49.8% (chest wall syndrome), 13.8 to 16.1% (cardiovascular diseases), 6.6 to 11.2% (stable coronary heart disease), 1.5 to 3.6% (acute coronary syndrome/myocardial infarction), 10.3 to 18.2% (respiratory diseases), 9.5 to 18.2% (psychogenic etiologies), 5.6 to 9.7% (gastrointestinal disorders), and 6.0 to 7.1% (esophageal disorders).

**Conclusion:**

This information may be of practical value for general practitioners as it provides the pre-test probabilities for a range of underlying diseases and may be suitable to guide the diagnostic process.

Chest pain is a common complaint in all health care settings and can be caused by a wide range of conditions – from diseases with favorable prognosis like musculoskeletal disorders to acute and potentially life-threatening conditions like coronary heart disease (1). Most patients with chest pain are initially seen by their general practitioner (GP) who faces the challenge to triage them. To fulfill this task, GPs need to know the relevant etiologies and their respective frequencies. In an intuitive process of probabilistic reasoning GPs combine the initial likelihood for a given etiology (pre-test probability) with their findings from the patient’s history and the clinical examination in order to reach a final or at least tentative diagnosis (post-test probability) (2,3).

Important information is provided by studies of symptoms, which investigate patients presenting with a defined symptom in a health care setting. In particular, they (4) aim to answer three main questions: How often do patients present with the respective symptom? What are the underlying conditions and their respective frequencies? What is the prognosis of these patients? While in the medical literature there are many studies on effects of treatment, causation of disease, or on diagnostic tests, studies of symptoms are not performed as often. As the results of single studies can show large variations, it is desirable to summarize existing information in a systematic review.

Therefore, we conducted a systematic review of studies investigating the symptom of chest pain in primary care. Since knowledge of relevant etiologies and their respective frequencies has the highest practical value for clinicians, we confine the current article to the reporting on this research question.

## Methods

### Search strategy and study selection

Eligible studies had to recruit unselected primary care patients presenting with chest pain as primary or secondary complaint. We excluded studies in which patients were recruited in secondary or tertiary health care settings. The studies had to recruit all chest pain patients regardless of the likelihood of a specific condition as the underlying etiology and had to report data on the frequency of at least one specific underlying condition.

We conducted comprehensive searches in PubMed (until October 2010) and EMBASE (until March 2011). We used search terms “chest pain” and “primary care.” Search strategies included subject headings (MeSH, Embtree) as well as free-text terms and were restricted to English and German (Supplementary material 1(web extra material 1)). We conducted a hand search in the online published abstracts of the annual meetings of the North American Primary Care Research Group and the European General Practice Research Network. We checked the reference lists of all relevant articles and asked experts in the field if they were aware of studies which were unpublished or ongoing.

Two reviewers independently screened all identified titles and abstracts for inclusion. If uncertainty remained, full-text articles were retrieved and comprehensively assessed for eligibility. Reviewers resolved any disagreements by consensus.

### Data extraction and quality assessment

One reviewer extracted data on study and patients’ characteristics and data on the frequencies of underlying etiologies following a pre-specified and standardized protocol. Currently there is no established approach to assess the risk of bias in studies of symptoms. We developed a risk of bias tool based on the sparse methodological literature (4,5) and own previous experience in the area (6,7). Two reviewers independently assessed the risk of bias separately for three key domains: selection of patients and GPs, data collection and patient flow, and determination of the underlying etiology. For each domain reviewers had to answer pre-specified and standardized signaling questions addressing relevant aspects of study design related to the potential of bias. The answers to these questions helped them to reach a judgement on the risk of bias in each domain. These were not, however, used as a score. A description of the risk of bias tool and details of the risk of bias assessment of the primary studies are available in Supplementary material 2(web extra material 2). In addition, we assessed whether study-specific inclusion criteria may have introduced clinical heterogeneity or variation, eg, we assumed that a study recruiting patients of all age groups would demonstrate different frequencies of the underlying conditions than a study recruiting patients aged >35 years.

### Analysis and data synthesis

We aimed to estimate how often chest pain was caused by a particular condition. We did not expect that all studies provided data on all diagnostic categories or conditions. For example, studies might have focused on one particular etiology or might have used definitions that did not match definitions used in other studies. Therefore, if a study did not provide data on a particular diagnostic category, we did not consider it in the analysis of this category rather than assuming a relative frequency of 0% with respect to that category. For each study presenting data for a particular condition we calculated the respective proportion and the 95% confidence interval using the Wilson procedure with a correction for continuity (8). We expected substantial between-study variation that is not due to chance. Variations in study design and risk of bias may cause methodological heterogeneity, while, eg, differences in inclusion criteria may cause clinical heterogeneity. To visualize variation across studies, we grouped all eligible studies by underlying conditions and plotted the results using forest plots. We used different measures to quantify the variability of probability estimates across studies. I^2^ quantifies the percentage of variation that is not due to chance (9). While its use is well established in meta-analyses of effects of interventions (9), its value is unclear in other kind of reviews. For example, it is not recommended to be used in diagnostic test accuracy reviews (10). Tau^2^ is an estimate of between-study variance in random-effects meta-analyses. In our case, the term “effect” refers to the proportion of patients with a particular condition. To estimate tau^2^, we used the restricted maximum likelihood method. The interpretation of tau^2^ is not very intuitive, but it is a measure that allows the calculation of a prediction interval. The “true” proportion of a future study that is similar to those included in the analysis will lie within the prediction interval with a probability of 95% (11). Besides the number of studies, the width of the interval is determined by the heterogeneity across studies. We believe that it is a more intuitive measure of heterogeneity. For the statistical computations and displays we used the statistical software R 3.1.1 (Foundation for Statistical Analysis, Vienna, Austria) and the package meta (12).

## Results

**Figure 1:**
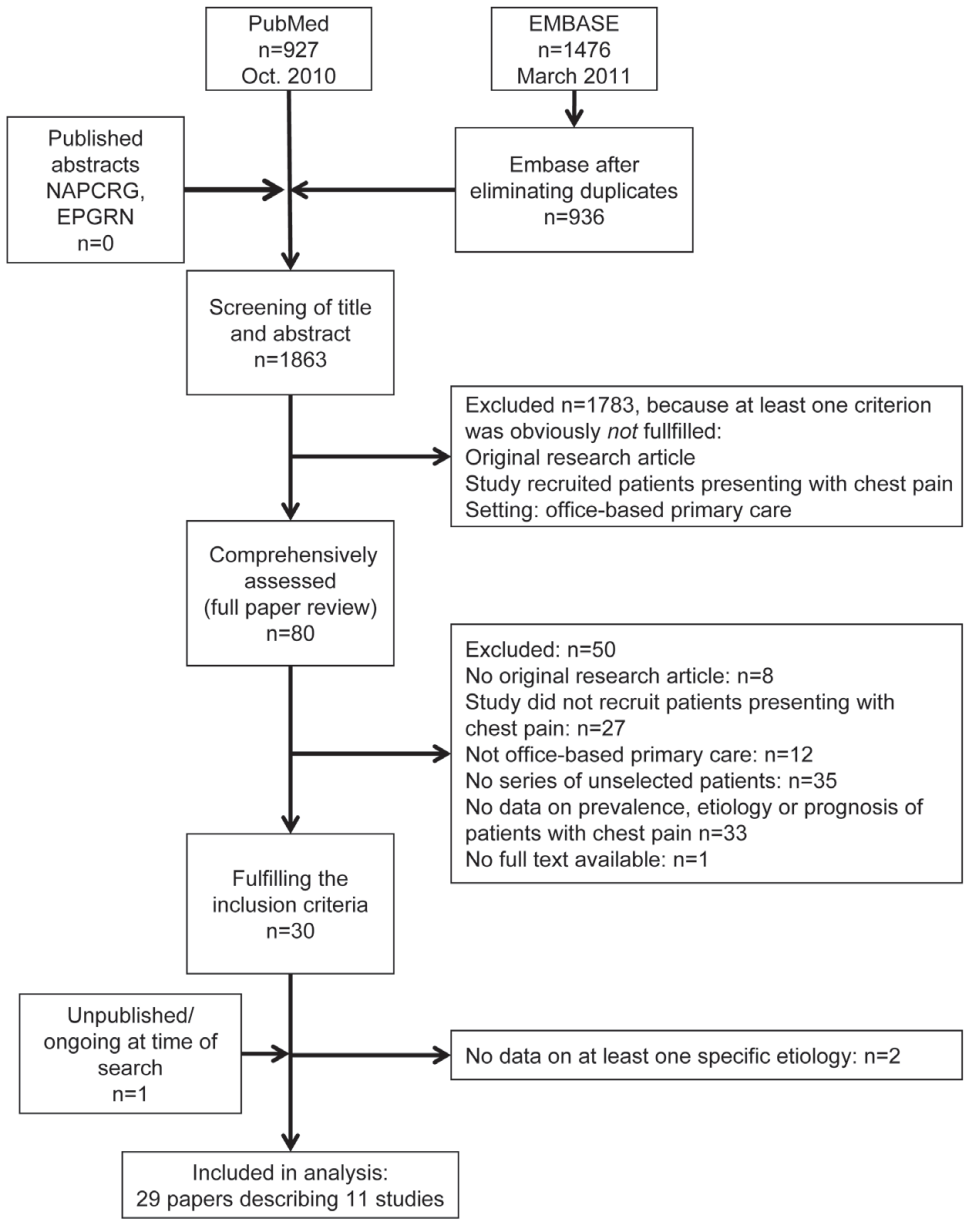
Search flow.

Our initial search identified 1863 references (Figure 1). After screening of titles and abstracts and comprehensive assessment of full papers we identified 31 references reporting data on 13 studies. One study reported data only on the prevalence of chest pain in primary care (13) and one study reported data only on two very broad categories of underlying conditions (organic etiology with and without signs) (14); both were therefore not considered in the current analysis. In total, we included 29 papers reporting data on 11 studies comprising about 6500 patients (Table 1). All studies were conducted in North-America or Europe between 1982 und 2010. The sex distribution across studies was reasonably homogeneous, with percentages of men in most studies ranging from 46% to 51%. In one small study (n = 51), the percentage of men was remarkably low (28%) (15). The studies varied somewhat with respect to the age limit. Five studies applied or reported no age limit (16-20). If reported, the percentage of children was low. In three studies the age limit varied between 16 and 20 years excluding children (21-23). Two studies included only patients aged ≥35 years (6,24). A detailed description of methodological characteristics of the included studies and the details of risk of bias assessment are available in Supplementary material 2(web extra material 2). In six studies we rated the risk of bias in all three key domains as low (Table 2).

**Table 1:** Characteristics of studies and patients

Study/ References*	Country	Time of data collection	Setting	Number of patients	Age (mean, standard deviation)^†^	Male sex,%	Inclusion/exclusion criteria
Rosser 1990 (16)	Canada	1985	109 general practitioners (GP) in 37 practices in 3 provinces	832	0-14: 1.2% 15-44: 34.1% 45-64: 32.6% 65+: 32.1	46.3	Chest pain as primary or secondary reason for encounter, no age limitation
Sox 1990 (17)	USA	1982	1 drop-in clinic	289	41 (n.r.)	51	Chest pain as presenting complaint, no age limitation (ages were 17 to 81 years). Patients were excluded from this sample if they had not had at least two chest pain episodes that led to the index visit or if they had a final diagnosis of myocardial infarction (MI).
Buntinx 1991 (18)	Belgium	1988	25 GPs	318	45 (19)	48	New episode of chest pain, discomfort or tightness as main or ancillary complaint, no age limitation
Klinkman 1994 (21)	USA	1992-93	11 primary care practices, Michigan	392	n.r^‡^	n.r^‡^	Adult patients who expressed chief complaint of chest pain or its equivalent. Only patients who were making their first visit in the particular episode were enrolled, patients seen elsewhere for an initial visit were excluded.
Svavarsdottir 1996 (19)	Iceland	1989-90	1 primary health care center	190	n.r.^§^	n.r.^§^	New episodes of chest pain, follow-up visits for the same episode were excluded, no age limitation mentioned, 6.3% were ≤20 years
Katerndahl 1997 (15)	USA	1994-95	8 family practice physicians, each from a different practice, South Texas	51	42.6 (14.6)	28	Patients with a new complaint of chest pain, 18 years and older.
Nilsson 2008 (22)	Sweden	1998-2000	3 health care centers each served by 4 GPs	516	54 (range 20-79)	49.6	New episode of chest pain, discomfort, or tightness as presenting complaint; aged 20-79 years; patients were excluded: if acute MI or coronary re-vascularization during the previous year
Verdon 2008 (TOPIC) (23)	Switzerland	2001	58 GPs in private practice	672	55 (19)	47.6	Chest pain as main or ancillary complaint; age ≥16 years
Bruyninkx 2009 (20)	Belgium	2003	GPs from all regions of the country covering almost 1.6% of the Belgian population	1996	58.6 (18.1)	51.6	Patients consulting their GPs with non-traumatic chest pain, no age limitation mentioned.
Bösner 2009 (6)	Germany	2004-05	74 GPs in private practice, located in the state of Hesse	1212	59 (-)	44.1	Chest pain as main or ancillary complaint; age ≥35 years; excluded: chest pain ≥1 one month, or had already been investigated
Haasenritter 2012 (24)	Germany	2009-10	56 GPs in private practice, located in the state of Hesse	856	59.5 (13.9)	48.5	Chest pain as main or ancillary complaint; age ≥35 years; excluded: chest pain ≥1 one month, or had already been investigated

*If several papers reported results on one study, we cited only the paper providing the most valuable information for the purpose of this review. However, all papers were comprehensively assessed and were cited in supplementary material 2(web extra material 2).

†Unless stated otherwise.

^‡^Authors stated that the age distribution of study patients closely approximated normal distribution, with a slight preponderance of younger adults, that slightly more women than men were included in the study, and that men were somewhat younger than women.

^§^Authors provided a figure displaying the age and sex distribution of patients. n.r.– not reported.

**Table 2:** Risk of bias

	Domain		
Study	Selection of patients/ general practitioners	Data collection	Diagnostic work up
Rosser 1990 (16)	low	low	high
Sox 1990 (17)	low	low	low
Buntinx 1991 (18)	low	low	low
Klinkmann 1994 (21)	low	low	unclear
Svavarsdottir 1996 (19)	high	unclear	unclear
Katerndahl 1997 (15)	low	low	high
Nilsson 2008 (22)	low	low	low
Verdon 2008 (23)	low	low	low
Bösner 2009 (6)	low	low	low
Bruyninckx 2009 (20)	low	low	high
Haasenritter 2012 (24)	low	low	low

The studies varied with respect to the number and definition of the considered underlying conditions. Three studies focused on coronary heart disease only (17,22,24). Among others, two studies provided data on the specific diagnoses of a wide range of underlying conditions (18,23), while six studies provided data mainly on broader diagnostic categories. In several studies, the only specific condition addressed was coronary heart disease (acute and stable). We considered the following diagnostic categories: cardiovascular, gastrointestinal, esophageal, respiratory, and psychogenic disorders, chest wall syndrome and trauma. In addition, we considered one specific disease (acute and stable coronary heart disease).

Supplementary material 3(web extra material 3) shows the forest plots for all diagnostic categories and conditions included in the analysis. For most of these diagnostic categories, we found substantial heterogeneity across studies indicated by high values of I^2^ and tau^2^ and by wide prediction intervals. Heterogeneity was in some cases moderately reduced by limiting the analysis to the studies with a low overall risk of bias (Table 3). Therefore, we decided to provide only a qualitative summary instead of pooled estimates.

**Table 3:** Relative frequencies and measures of heterogeneity of different underlying conditions of chest pain in primary care considering only studies with a low overall risk of bias

Study	N	Percentage	95% confidence interval (CI)
Coronary heart disease (any)			
Buntinx 1991 (18)	318	9.7	6.8%-13.7%
Nilsson 2008 (22)	516	11.8	9.2%-15.0%
Verdon 2008 (23)	672	12.6	10.3%-15.5%
Bösner 2009 (6)	1212	14.8	12.8%-16.9%
Haasenritter 2012 (24)	856	10.9	8.9%-13.2%
Minimum-maximum	9.7%-14.8%		
I^2^	60.4% (95% CI: 0.0%-85.2%)		
Tau^2^	0.02		
Prediction interval	7.7%-18.8%		
Coronary heart disease (stable)			
Sox 1990 (17)	289	8.0	5.2%-11.9%
Buntinx 1991 (18)	318	6.6	4.2%-10.1%
Verdon 2008 (23)	672	11.2	8.9%-13.8%
Bösner 2009 (6)	1212	11.1	9.5%-13.1%
Haasenritter 2012 (24)	856	8.3	6.6%-10.4%
Minimum-maximum	6.6%-11.2%		
I^2^	62.8% (95% CI: 1.6%-86.0%)		
Tau^2^	0.03		
Prediction interval	4.9%-16.8%		
Acute coronary syndrome/myocardial infarction			
Buntinx 1991 (18)	318	3.1	1.6%-5.9%
Verdon 2008 (23)	672	1.5	0.8%-2.8%
Bösner 2009 (6)	1212	3.6	2.7%-4.9%
Haasenritter 2012 (24)	856	2.6	1.7%-3.9%
Minimum-maximum	1.5%-3.6%		
I^2^	58.6% (95% CI: 0.0%-86.2%)		
Tau^2^	0.08		
Prediction interval	0.6%-10.6%		
Cardiovascular diseases			
Buntinx 1991 (18)	318	13.8	10.3%-18.2%
Verdon 2008 (23)	672	16.1	13.4%-19.1%
Minimum-maximum	13.8%-16.1%		
I^2^	0% (95% CI: NA)		
Tau^2^	0		
Prediction interval	NA		
Gastrointestinal disorders			
Buntinx 1991 (18)	318	9.7	6.8%-13.7%
Verdon 2008 (23)	672	8.2	6.3%-10.6%
Bösner 2009 (6)	1212	5.6	4.4%-7.1%
Minimum-maximum	5.6%-9.7%		
I^2^	76.7% (95% CI: 23.9%-92.9%)		
Tau^2^	0.07		
Prediction interval	0.1%-82.6%		
Esophageal disorders			
Buntinx 1991 (18)	318	6.0	3.7%-9.3%
Verdon 2008 (23)	672	7.1	5.4%-9.4%
Minimum-maximum	6.0%-7.1%		
I^2^	0% (95% CI: NA)		
Tau^2^	0		
Prediction interval	NA		
Respiratory diseases			
Buntinx 1991 (18)	318	18.2	14.2%-23.0%
Verdon 2008 (23)	672	10.3	8.1%-12.9%
Bösner 2009 (6)	1212	12.0	10.3%-14.0%
Minimum-maximum	10.3%-18.2%		
I^2^	84.2% (95% CI: 52.8%-94.7%)		
Tau^2^	0.10		
Prediction interval	0.1%-94.0%		
Psychogenic			
Buntinx 1991 (18)	318	18.2	14.2%-23.0%
Verdon 2008 (23)	672	11.5	9.2%-14.2%
Bösner 2009 (6)	1212	9.5	7.9%-11.3%
Minimum-maximum	9.5%-18.2%		
I^2^	89.3% (95% CI: 70.8%-96.0%)		
Tau^2^	0.13		
Prediction interval	0.1%-97.0%		
Chest wall syndrome			
Buntinx 1991 (18)	318	24.5	20.0%-29.7%
Verdon 2008 (23)	672	48.8	45.0%-52.7%
Bösner 2009 (6)	1212	49.8	47.0%-52.7%
Minimum-maximum	24.5%-49.8%		
I^2^	96.9% (95% CI: 93.6%-98.5%)		
Tau^2^	0.38		
Prediction interval	0.0%-100.0%		
Trauma			
Verdon 2008 (23)	672	3.9	2.6%-5.7%
Bösner 2009 (6)	1212	3.2	2.3%-4.4%
Haasenritter 2012(24)	856	1.8	1.0%-2.9%
Minimum-maximum	1.8%-3.9%		
I^2^	68.6% (95% CI: 0.0%-90.9%)		
Tau^2^	0.11		
Prediction interval	0.0%-83.0%		

Table 3 provides the results of the studies with a low overall risk of bias. We found that myocardial ischemia was the underlying condition of chest pain in 9.7 to 14.8% of chest pain cases. Stable CHD caused chest pain in 6.6%-11.2% of cases and acute coronary syndrome (ACS) or myocardial infarction (MI) in 1.5%-3.6% of cases. The relative frequencies of other conditions ranged from 24.5 to 49.8% (chest wall syndrome), 13.8 to 16.1% (cardiovascular diseases), 10.3 to 18.2% (respiratory diseases), 9.5 to 18.2% (psychogenic etiologies), 5.6 to 9.7% (gastrointestinal disorders), and 6.0 to 7.1% (esophageal disorders)

## Discussion

This systematic review identified 11 eligible studies investigating the causes of chest pain in the primary care setting comprising about 6500 patients. However, only 6 studies, comprising about 3900 patients, were methodologically sound and therefore appropriate to inform clinical practice.

To our best knowledge, this is the first review that systematically investigated the symptom of chest pain in primary care. Strengths of our study are the comprehensive search and the rigorous assessment of the risk of bias. Its limitations are the small number of studies and the heterogeneity across studies. Besides methodological reasons, this may be caused by different definitions of the diagnostic categories. However, our study gave important insight into the frequencies of relevant causes of chest pain in primary care and may be helpful for clinicians. Although they most likely do not deliberately reflect on it, GPs in their approach to chest pain patients apply probabilistic or Bayesian reasoning (2). In order to start the process of Bayesian arguing, they have to know the pre-test probabilities of different differential diagnoses.

The current review focuses on studies conducted in primary care. Our findings principally confirmed the results of Buntinx et al (25), who showed that there was a large difference in the diagnostic case mix presented in general practice compared with emergency departments or secondary care. In a previous systematic review on the accuracy of symptoms and signs for CHD we included 172 studies (26). The overwhelming majority of these studies recruited patients presenting with chest pain in secondary care or emergency departments. The percentage of cases with stable CHD as underlying condition was 52% (median) and the percentage of cases with ACS or MI as underlying condition was 37% (median). The relative frequencies of stable CHD and ACS/MI reported in primary care were distinctly lower.

Another reason why there is a need for robust data to describe the distribution for pre-test probabilities in chest pain patients is the fact that the diagnostic accuracy of consequently applied tests seems to vary with the underlying case mix (27). When they compared patients with chest pain in two high- and two low-disease prevalence populations, Sox et al (17) showed that patient history as a diagnostic test to estimate the probability of CHD did not show the same validity in both settings. Test accuracy of patient history and corresponding post-test probabilities for CHD depended on the prior probability of disease. These findings are supported by Knottnerus et al (28), who showed that the setting where a study was conducted influenced the characteristics of diagnostic tests. Therefore, it is important to provide exact data that reflect the different spectrum of disease in chest pain patients in primary care compared to the emergency department.

In conclusion, this review provided data on relative frequencies of several causes of chest pain in primary care. This knowledge may guide the initial diagnostic reasoning of clinicians when approaching chest pain patients in primary care. Because of unexplained heterogeneity, however, clinicians should use our results with caution. There is a need for large and methodologically sound studies investigating common symptoms in primary care. Ideally, these studies would not only determine the relative frequencies of all relevant differential diagnoses, but also investigate the diagnostic accuracy of symptoms, signs, and point-of-care tests considering the whole spectrum of relevant target diseases (29). Previously, a design for this kind of studies was suggested and discussed (30). The results could inform primary care health professionals how to effectively assess and triage patients presenting with particular symptoms.
