# Supplementary material for: Causes of chest pain in primary care – a systematic review and meta-analysis
Source: Croat Med J. 2015 Oct;56(5):422–30. doi: 10.3325/cmj.2015.56.422 (PMC4655927; doi:10.3325/cmj.2015.56.422)
Supplement: Supplementary Material 1 [file CroatMedJ_56_s001.pdf]

Supplement 1: Search strategy (Pubmed)

<table>
<tr><td>chest [TIAB]
OR
thoracic [TIAB]
OR
thorax[TIAB]</td><td>AND</td><td>pain[TIAB]
OR
pains[TIAB]
OR
discomfort[TIAB]
OR
complaint*[TIAB]
OR
distress*[TIAB]
OR
pressure*[TIAB]
OR
numbness[TIAB]</td></tr>
<tr><td colspan="3">OR</td></tr>
<tr><td colspan="3">"chest pain"[Mesh]</td></tr>
<tr><td colspan="3">AND</td></tr>
<tr><td colspan="2">"general practitioner” [TIAB]
“general practitioners”[TIAB]
“general practice” [TIAB]
“family practice”[TIAB]
“family practitioners” [TIAB]
“family practitioner” [TIAB]
“family medicine” [TIAB]
“family physician” [TIAB]
“family physicians” [TIAB]
“family doctor” [TIAB]
“family doctors” [TIAB]
“primary care” [TIAB]</td><td>OR</td></tr>
<tr><td colspan="3">OR</td></tr>
<tr><td colspan="2">"BMC Fam Pract"[TA]
"Fam Pract"[TA]
"J Fam Pract"[TA]
"Fam Pract Res J"[TA]
"J Am Board Fam Pract"[TA]
"Br j gen pract"[TA]
“J R Coll Gen Pract” [TA]
“J Coll Gen Pract” [TA]
“J Coll Gen Pract Res Newsl”[TA]
"Can fam physician"[TA]
"Ann Fam Med"[TA]
"Aust fam physician"[TA]
"Scand J Prim Health Care"[TA]
"Eur J Gen Pract"[TA]
"Archives of family medicine"[Journal]
“J Gen Intern Med”[TA]
"Atencion primaria / Sociedad Española de Medicina de Familia y Comunitaria"[Journal]</td><td>OR</td></tr>
<tr><td colspan="3">OR</td></tr>
<tr><td colspan="2">“general practice” [AD]
“family practice*” [AD]
“family medicine” [AD]
“primary care” [AD]
community [AD]</td><td>OR</td></tr>
<tr><td colspan="3">OR</td></tr>
</table>

"Family Practice"[Mesh]<br>"Physicians, Family"[Mesh]<br>"Primary Health Care"[Mesh]<br>“Community Health Service”[Mesh]<br>“Rural Health Service”[Mesh]<br>“Rural Health”[Mesh]	OR

Limits: NOT: Editorial, Meta-Analysis, Practice Guideline, Review, Addresses, Bibliography, Biography, Case Reports, Comment, Dictionary, Directory, Festschrift, Government Publications, Guideline, Historical Article, In Vitro, Interactive Tutorial, Interview, Legal Cases, Legislation, Patient Education Handout, Portraits, Webcasts
