# Supplementary material for: Causes of chest pain in primary care – a systematic review and meta-analysis
Source: Croat Med J. 2015 Oct;56(5):422–30. doi: 10.3325/cmj.2015.56.422 (PMC4655927; doi:10.3325/cmj.2015.56.422)
Supplement: Supplementary Material 1 [file CroatMedJ_56_s002.pdf]

Supplement 2: Risk of Bias

2.1 Risk of Bias Tool

The following table gives an overview of the “Risk of Bias Tool” that we used to assess the risk of bias. For each domain, reviewers answered signalling question and assessed the risk of bias. In the first domain, reviewers also rated their concern, that the selection of patients (and GPs) introduced substantial variation:

Domain A: Selection of patients and GPs (refers to all studies regardless the review question)		
Item/ Signalling Question		Refers to
1	Was the symptom to be investigated clearly described?	Information
2	Were the selection criteria of the patients clearly described?	Information
3	Was a consecutive or random sample of patients enrolled?	Bias
4	Was it a multi-centre study?	Bias
5	Did the selection criteria of the patients permit the study population to represent the full spectrum of those presenting with the symptom in the respective setting?	Variation
6	Were the participating health care professionals/ institutions representative for the setting to be investigated in the review?	Variation
Concern that the selection of patients introduced substantial variation: low, unclear, high		
Risk that the selection of patients introduced bias: low, unclear, high		
Domain B: Data collection and patient flow (refers to all studies regardless of the review question)		
Item/ Signalling Question		Refers to
7	Were data about the symptom und the inclusion criteria collected directly from the patients (as opposed to a proxy like a register, routine documentation)	Bias
8	Was the same mode of data collection used for all patients?	Bias
9	Was the number of non-responders/ dropouts unlikely to affect the results?	Bias
Risk that the mode of data collection and/ or patient flow introduced bias: low, unclear, high		
Domain C: Determination of the underlying etiology of the symptom (refers only to review question “What are the underlying conditions and their respective frequencies (differential diagnosis)?”)		
Item/ Signalling Question (to be answered for every etiologic category reported in a study)		Refers to
10	Was the etiologic category clearly defined?	Information
11	Was the diagnostic work up likely to correctly classify the respective aetiology?	Bias
12	Did every patient receive the same diagnostic work up to detect the respective etiology?	Bias
Risk that the diagnostic work up introduce bias: low, unclear, high		

2.2 Assessment of included studies

Study ID: Rosser 1990 [1]

**Domain A: Selection of patients and GPs**		
Risk that the selection of patients introduced bias?		low
Concern that the selection of patients introduced substantial variation?		low
Support for judgement		
I1	Was the symptom to be investigated clearly described?	no
I2	Were the selection criteria of the patients clearly described?	yes
I3	Was a consecutive or random sample of patients enrolled?	yes
I4	Was it a multi-centered study?	yes
I5	Did the selection criteria of the patients permit the study population to represent the full spectrum of those presenting with the symptom in the respective setting?	yes
I6	Were the participating health care professionals/ institutions representative for the setting to be investigated in the review?	yes
Symptom: No definition provided<br>Recruitment: For 13 consecutive weeks the clinicians recorded data about each consultation with patients in their own practice during which chest pain was discussed, investigated, or treated in a face-to-face encounter (excluding patients already hospitalized).<br>Additional comments:<br>GPs: One hundred nine clinicians in 37 practices in 18 states and three Canadian provinces took part (members of the Ambulatory Sentinel Practice Network).<br>If the same patient was reported more than one during the study period, only data from the first visit were reported and analyzed.<br>Patients: No age limit or other in- or exclusion criteria mentioned; children presented 1% of patients presenting with chest pain.		
**Domain B: Data collection and patient flow**		
Risk that the mode of data collection and/ or patient flow introduced bias		low
Support for judgement		
I7	Were data about the symptom und the inclusion criteria collected directly from the patients (as opposed to a proxy like a register, routine documentation)	yes
I8	Was the same mode of data collection used for all patients?	yes
I9	Was the number of non-responders/ dropouts unlikely to affect the results?	unclear
Additional comments: prospective data collection; no drop-outs mentioned.		
**Domain C: Determination of the underlying etiology of the symptom**		
Risk that the diagnostic work up introduce bias?		high
Support for judgement:		
I10	Was the etiologic category clearly defined?	no
I11	Was the diagnostic work up likely to correctly classify the respective etiology?	no
I12	Did every patient receive the same diagnostic work up to detect the respective etiology?	no
Additional comments: Diagnostic work-up and the approach how the diagnosis were established are not described. No systematic follow-up mentioned; at least in a part of the patients the		

reported diagnosis seems to be based solely on the initial judgement.

Study ID: Sox 1990 [2]

**Domain A: Selection of patients and GPs**		
Risk that the selection of patients introduced bias?		low
Concern that the selection of patients introduced substantial variation?		unclear
Support for judgement		
I1	Was the symptom to be investigated clearly described?	no
I2	Were the selection criteria of the patients clearly described?	yes
I3	Was a consecutive or random sample of patients enrolled?	yes
I4	Was it a multi-centered study?	no
I5	Did the selection criteria of the patients permit the study population to represent the full spectrum of those presenting with the symptom in the respective setting?	unclear
I6	Were the participating health care professionals/ institutions representative for the setting to be investigated in the review?	yes
Additional comments: Symptom: no definition provided;<br>Patients and GPs: all chest pain patients were seen in the drop-in clinic or emergency room of Kaiser-Permanente Center, Santa Clara, California. No age or other limits mentioned:		
**Domain B: Data collection and patient flow**		
Risk that the mode of data collection and/ or patient flow introduced bias		low
Support for judgement		
I7	Were data about the symptom und the inclusion criteria collected directly from the patients (as opposed to a proxy like a register, routine documentation)	yes
I8	Was the same mode of data collection used for all patients?	yes
I9	Was the number of non-responders/ dropouts unlikely to affect the results?	yes
Additional comments: prospective data collection; exclusion of patients (194) who were nearly all outpatients whose index visit was for a first episode of chest pain or whose final diagnosis was acute MI.		
**Domain C: Determination of the underlying etiology of the symptom**		
Risk that the diagnostic work up introduce bias?		low
Support for judgement:		
I10	Was the etiologic category clearly defined?	yes
I11	Was the diagnostic work up likely to correctly classify the respective etiology?	yes
I12	Did every patient receive the same diagnostic work up to detect the respective etiology?	no
Additional comments: Each patient had one follow-up interview by a research assistant who inquired about clinical outcome and subsequent care. The median time to the follow-up interview was 20 days. Each patient was assigned a clinical diagnosis by two physicians who independently reviewed all study data and the patient's medical record. The reviews were conducted at least 1 year after the index visit. Standard clinical criteria for angina pectoris, coronary insufficiency, and acute myocardial infarction were those used in the Coronary Drug Project. Although these criteria		

were made available to the physicians, they were free to incorporate all information, including diagnostic test results, clinical outcome, and the opinion of the physicians who cared for the patient, into their diagnostic judgment. The physicians agreed on a diagnosis of CAD in 90.2% of the VA patients (kappa = 0.80, *P* <0.0001 that agreement was due to chance). When the physicians disagreed on the diagnosis in a VA patient, two other physicans reviewed the record, and the majority opinion was used. The physicians agreed on a diagnosis of CAD in 94.8% of the VA patients (kappa = 0.80, *P* <0.0001 that agreement was due to chance). When the physicians disagreed on the diagnosis in a Kaiser patient, they discussed the case and reached a consensus diagnosis.

Study ID: Buntinx 1991 [3–5]

**Domain A: Selection of patients and GPs**		
Risk that the selection of patients introduced bias?		low
Concern that the selection of patients introduced substantial variation?		low
Support for judgement		
I1	Was the symptom to be investigated clearly described?	no
I2	Were the selection criteria of the patients clearly described?	yes
I3	Was a consecutive or random sample of patients enrolled?	yes
I4	Was it a multi-centered study?	yes
I5	Did the selection criteria of the patients permit the study population to represent the full spectrum of those presenting with the symptom in the respective setting?	yes
I6	Were the participating health care professionals/ institutions representative for the setting to be investigated in the review?	yes
Additional comments: Symptom: no definition provided;<br>GPs: 25 GPs in Belgium, practices were spread over the Flemish part of Belgium<br>Patients and Recruitment: All patients who complained to their GP of a new episode of chest pain, discomfort or tightness were included.(February and March 1988)		
**Domain B: Data collection and patient flow**		
Risk that the mode of data collection and/ or patient flow introduced bias		low
Support for judgement		
I7	Were data about the symptom und the inclusion criteria collected directly from the patients (as opposed to a proxy like a register, routine documentation)	yes
I8	Was the same mode of data collection used for all patients?	yes
I9	Was the number of non-responders/ dropouts unlikely to affect the results?	yes
Additional comments: Data were collected prospectively; after each contact with the patient GPs completed a questionnaire. No drop outs mentioned.		
**Domain C: Determination of the underlying etiology of the symptom**		
Risk that the diagnostic work up introduce bias?		low
Support for judgement:		
I10	Was the etiologic category clearly defined?	yes
I11	Was the diagnostic work up likely to correctly classify the respective etiology?	yes
I12	Did every patient receive the same diagnostic work up to detect the respective etiology?	no

Additional comments: At least 2 weeks (maximum 2 months) after the first contact, a second report was completed, containing the follow-up diagnosis, using all data now available from technical examinations, from referrals or from the further evolution of the disease. The diagnosis of this follow-up registration was considered as final diagnosis, used as the comparing standard or best possible diagnosis. No previously defined diagnostic standards or protocols were used. Each practitioner followed his usual diagnostic criteria. Diagnosis were classified according to the International Classification of Primary Care.

Study ID: Klinkman 1994 [6]

**Domain A: Selection of patients and GPs**		
Risk that the selection of patients introduced bias?		low
Concern that the selection of patients introduced substantial variation?		unclear
Support for judgement		
I1	Was the symptom to be investigated clearly described?	no
I2	Were the selection criteria of the patients clearly described?	yes
I3	Was a consecutive or random sample of patients enrolled?	yes
I4	Was it a multi-centered study?	yes
I5	Did the selection criteria of the patients permit the study population to represent the full spectrum of those presenting with the symptom in the respective setting?	unclear
I6	Were the participating health care professionals/ institutions representative for the setting to be investigated in the review?	yes
Additional comments: Symptom: no specific definition provided;<br>Patients and recruitment: The study enrolled all adult patients who expressed the chief complaint of "chest pain" or its equivalent to the office staff at the time of entry to the office or to the medical staff at the time of entry into the examination room. Only those patients who were making their first visit for this particular episode of chest pain were enrolled in the study; patients seen elsewhere for an initial visit were excluded. This restriction was designed to create a data set containing episodes of "non-emergent" chest pain, followed from the beginning of the episode of care. No age limitation mentioned,<br>GPs: Eleven practices of a research network (IRNET) in Michigan, USA, data were collected between January 1992 and March 1993		
**Domain B: Data collection and patient flow**		
Risk that the mode of data collection and/ or patient flow introduced bias		low
Support for judgement		
I7	Were data about the symptom und the inclusion criteria collected directly from the patients (as opposed to a proxy like a register, routine documentation)	yes
I8	Was the same mode of data collection used for all patients?	yes
I9	Was the number of non-responders/ dropouts unlikely to affect the results?	unclear
Additional comments: Prospective data collection; Clinicians were asked to complete a visit form at the time of initial and all follow-up office visits during an episode of "chest pain." The information collected can be described by categories: demographic information: age, sex, insurance type; "Physician decision-making" factors: familiarity with patient, prior history of chest pain or related disease, acute versus chronic pain, severity of pain; Utilization information: length of visit, laboratory, ancillary, referral data; Diagnosis information: Diagnosis(es) made, confidence in diagnosis; Disposition information: admission to hospital, follow-up care, off-work information.		

Three methods for data validation (To search for selection bias in the episodes captured for the study; to confirm the validity of clinician data recording and completeness of captured episodes; to estimate the accuracy of the coding and data entry process) were applied.

**Domain C: Determination of the underlying etiology of the symptom**		
Risk that the diagnostic work up introduce bias?		unclear
Support for judgement:		
I10	Was the etiologic category clearly defined?	no
I11	Was the diagnostic work up likely to correctly classify the respective etiology?	unclear
I12	Did every patient receive the same diagnostic work up to detect the respective etiology?	no
Additional comments: Follow-up diagnosis of the GPs were used to establish the final diagnosis; no systematic follow up of patients, at least one part of the final diagnoses is based simply on the initial diagnosis of the GPs, (further details see Domain B)		

Study ID: Svavarsdottir 1996 [7]

**Domain A: Selection of patients and GPs**		
Risk that the selection of patients introduced bias?		high
Concern that the selection of patients introduced substantial variation?		unclear
Support for judgement		
I1	Was the symptom to be investigated clearly described?	no
I2	Were the selection criteria of the patients clearly described?	unclear
I3	Was a consecutive or random sample of patients enrolled?	unclear
I4	Was it a multi-centered study?	no
I5	Did the selection criteria of the patients permit the study population to represent the full spectrum of those presenting with the symptom in the respective setting?	yes
I6	Were the participating health care professionals/ institutions representative for the setting to be investigated in the review?	yes
Additional comments: Symptom: no specific definition provided;<br>Patients: no age limitation or other in- or exclusion criteria mentioned;<br>GPs: 1 health care center in Reykjavik City, Iceland		
**Domain B: Data collection and patient flow**		
Risk that the mode of data collection and/ or patient flow introduced bias		unclear
Support for judgement		
I7	Were data about the symptom und the inclusion criteria collected directly from the patients (as opposed to a proxy like a register, routine documentation)	no
I8	Was the same mode of data collection used for all patients?	unclear
I9	Was the number of non-responders/ dropouts unlikely to affect the results?	unclear
Additional comments: retrospective data collection using routine data		
**Domain C: Determination of the underlying etiology of the symptom**		
Risk that the diagnostic work up introduce bias?		unclear
Support for judgement:		

I10	Was the etiologic category clearly defined?	no
I11	Was the diagnostic work up likely to correctly classify the respective etiology?	unclear
I12	Did every patient receive the same diagnostic work up to detect the respective etiology?	no
Additional comments: Unclear which approach was used to reach a final diagnosis; follow up after 3 years was used to answer 3 questions regarding the course of chest pain; plots include some mistakes (labels of the x-axis)		

Study ID: Katerndahl 1997 [8]

**Domain A: Selection of patients and GPs**		
Risk that the selection of patients introduced bias?		low
Concern that the selection of patients introduced substantial variation?		unclear
Support for judgement		
I1	Was the symptom to be investigated clearly described?	no
I2	Were the selection criteria of the patients clearly described?	yes
I3	Was a consecutive or random sample of patients enrolled?	yes
I4	Was it a multi-centered study?	yes
I5	Did the selection criteria of the patients permit the study population to represent the full spectrum of those presenting with the symptom in the respective setting?	yes
I6	Were the participating health care professionals/ institutions representative for the setting to be investigated in the review?	yes
Additional comments: Symptom: no specific definition mentioned<br>Patients and recruitment: Between June 1994 and October 1995, the office staff in each STARNET practice identified consecutive English-speaking adults 18 years and older who presented to the physician's office with a chief complaint of new-onset chest pain. This included patients with only one complaint (chest pain) as well as those with several symptoms that included chest pain. Patients were excluded if they had been seen previously for chest pain at the practice.<br>GPs: 8 family practice physicians participating in the South Texas Ambulatory Research Network (STARNET).		
**Domain B: Data collection and patient flow**		
Risk that the mode of data collection and/ or patient flow introduced bias		low
Support for judgement		
I7	Were data about the symptom und the inclusion criteria collected directly from the patients (as opposed to a proxy like a register, routine documentation)	yes
I8	Was the same mode of data collection used for all patients?	yes
I9	Was the number of non-responders/ dropouts unlikely to affect the results?	yes
Additional comments: not clear if, prospective data collection; 2 out of 53 patients declined		
**Domain C: Determination of the underlying etiology of the symptom**		

Risk that the diagnostic work up introduce bias?		high
Support for judgement:		
I10	Was the etiologic category clearly defined?	no
I11	Was the diagnostic work up likely to correctly classify the respective etiology?	no
I12	Did every patient receive the same diagnostic work up to detect the respective etiology?	no
Additional comments: Initial diagnoses of the GPs were provided		

Study ID: Nilsson 2008 [9–11]

Domain A: Selection of patients and GPs		
Risk that the selection of patients introduced bias?		low
Concern that the selection of patients introduced substantial variation?		low
Support for judgement		
I1	Was the symptom to be investigated clearly described?	no
I2	Were the selection criteria of the patients clearly described?	yes
I3	Was a consecutive or random sample of patients enrolled?	yes
I4	Was it a multi-centered study?	yes
I5	Did the selection criteria of the patients permit the study population to represent the full spectrum of those presenting with the symptom in the respective setting?	yes
I6	Were the participating health care professionals/ institutions representative for the setting to be investigated in the review?	yes
Additional comments: Symptom: 'Pain' was defined as pressure, ache, burning or a stabbing sensation, no specific definition regard location or duration (chronic or acute) provided;<br>Patients: During a 21-month period, from 1998 to 2000, consecutive patients with chest pain were investigated. The patients were 20 to 79 years old, no other limitations were mentioned; patients consulted their GP for a new episode of chest pain. ''New'' was defined as having commenced during the past 6 months and with a free interval of at least 6 months after any previous episode of the same type of complaint. All inclusions were made by the GP. Patients who, prior to the study, had been diagnosed as having coronary insufficiency by physiological methods were excluded. Those who had had an acute myocardial infarction or had been the subject of coronary revascularization during the previous year were also excluded.<br>GPs: 25 GPs in three neighbouring primary healthcare centers, with a listed population of 16 152 individuals aged 20-79 years, in the county of Östergötland		
**Domain B: Data collection and patient flow**		
Risk that the mode of data collection and/ or patient flow introduced bias		low
Support for judgement		
I7	Were data about the symptom und the inclusion criteria collected directly from the patients (as opposed to a proxy like a register, routine documentation)	yes
I8	Was the same mode of data collection used for all patients?	yes

I9	Was the number of non-responders/ dropouts unlikely to affect the results?	yes
Additional comments: Prospective data collection; thirty-eight patients were excluded from the analysis, reasons were reported		
**Domain C: Determination of the underlying etiology of the symptom**		
Risk that the diagnostic work up introduce bias?		low
Support for judgement:		
I10	Was the etiologic category clearly defined?	yes
I11	Was the diagnostic work up likely to correctly classify the respective etiology?	yes
I12	Did every patient receive the same diagnostic work up to detect the respective etiology?	yes
Additional comments Patients in whom IHD was judged to be excluded after a basic clinical examination were further managed according to normal clinical practice outside the study. They were allocated to the group ‘wait and see’’ in the analyses of GP action in daily practice. Patients in whom unstable IHD could not be ruled out were referred acutely to hospital. All remaining patients were referred for exercise testing. Three months after inclusion, a postal questionnaire was sent to those patients in whom IHD was judged to be excluded after a basic clinical examination. In the questionnaire, any physician-made diagnosis of angina pectoris or myocardial infarction after inclusion date was explored. Validation of affirmative replies was done retrospectively through the healthcare centers’ medical records. In addition, the medical records of patients not responding to the questionnaire were examined for possible IHD diagnosis. All remaining patients were referred either acutely to hospital or for exercise testing. The hospital investigation results were classified as ‘‘IHD’’ or ‘‘not IHD’’, based on the medical records. If diagnostic uncertainty remained after hospital investigation, exercise testing within the study was possible. If the result of the exercise test was equivocal, myocardial perfusion scintigraphy, using technetium 99-tetrofosmin (Myoview), was undertaken. The results after exercise testing or myocardial perfusion scintigraphy were classified as ‘‘IHD’’ or ‘‘not IHD”.		

Study ID: Bruyninkx 2009 [12–14]

**Domain A: Selection of patients and GPs**		
Risk that the selection of patients introduced bias?		low
Concern that the selection of patients introduced substantial variation?		low
Support for judgement		
I1	Was the symptom to be investigated clearly described?	no
I2	Were the selection criteria of the patients clearly described?	yes
I3	Was a consecutive or random sample of patients enrolled?	yes
I4	Was it a multi-centered study?	yes
I5	Did the selection criteria of the patients permit the study population to represent the full spectrum of those presenting with the symptom in the respective setting?	yes
I6	Were the participating health care professionals/ institutions representative for the setting to be investigated in the review?	yes

Additional comments: Symptom: no specific definition provided<br>Patients: All patients consulting their GP with non-traumatic chest pain in 2003 were consecutively included in the study. No age or other limitations mentioned<br>GPs: The study was carried out in the Belgian sentinel network of general practices. GPs: n=163; This network has been established 25 years ago as a voluntary and permanent registry of epidemiological data. The network consists of GPs of all regions of the country and is representative with respect to gender and age. Only physicians regularly recording patients with chest pain for 26 or more weeks participated in the study. During this study period, the network covered almost 1.6% of the Belgian population.		
**Domain B: Data collection and patient flow**		
Risk that the mode of data collection and/ or patient flow introduced bias		low
Support for judgement		
I7	Were data about the symptom und the inclusion criteria collected directly from the patients (as opposed to a proxy like a register, routine documentation)	yes
I8	Was the same mode of data collection used for all patients?	yes
I9	Was the number of non-responders/ dropouts unlikely to affect the results?	yes
Additional comments: Prospective data collection; At the time of consultation, the patient's gender and age, and the GP's initial diagnosis, degree of certainty of the initial diagnosis and action taken were recorded on special forms; 24 patients were excluded from the analysis, reasons were provided		
**Domain C: Determination of the underlying etiology of the symptom**		
Risk that the diagnostic work up introduce bias?		high
Support for judgement:		
I10	Was the etiologic category clearly defined?	no
I11	Was the diagnostic work up likely to correctly classify the respective etiology?	no
I12	Did every patient receive the same diagnostic work up to detect the respective etiology?	no
Additional comments: Initial diagnosis of the GPs were reported. How diagnoses were made was left to the discretion of the treating physicians.		

Study ID: Bösner 2009 [15–24]

**Domain A: Selection of patients and GPs**		
Risk that the selection of patients introduced bias?		low
Concern that the selection of patients introduced substantial variation?		unclear
Support for judgement		
I1	Was the symptom to be investigated clearly described?	yes
I2	Were the selection criteria of the patients clearly described?	yes
I3	Was a consecutive or random sample of patients enrolled?	yes
I4	Was it a multi-centered study?	yes
I5	Did the selection criteria of the patients permit the study population to represent the full spectrum of those presenting with the symptom in the respective setting?	unclear

<table>
<tr><td>I6</td><td>Were the participating health care professionals/ institutions representative for the setting to be investigated in the review?</td><td>yes</td></tr>
<tr><td colspan="3">Additional comments: Symptom: pain, tightness, or oppression localized in the area between the clavicles and lower costal margins and anterior to the posterior axillary lines, acute or chronic
Patients and recruitment: Every patient above 35 years with a complaint of chest pain was to be included. Doctors were also asked to recruit at home visits and emergency calls. Patients were eligible irrespective of the acute or chronic nature of their complaint, or of previously known conditions including IHD or related risk factors. Patients whose chest pain had subsided for more than 1 month, whose chest pain had been investigated already, and/or who came for follow-up for chest pain were excluded.
GPs: 69 GPs in state of Hesse, Germany; details on GPs' characteristic (age, sex, experience in years, practice location) were provided.</td></tr>
<tr><td colspan="3"></td></tr>
<tr><td colspan="3">Domain B: Data collection and patient flow</td></tr>
<tr><td colspan="2">Risk that the mode of data collection and/ or patient flow introduced bias</td><td>low</td></tr>
<tr><td colspan="3">Support for judgement</td></tr>
<tr><td>I7</td><td>Were data about the symptom und the inclusion criteria collected directly from the patients (as opposed to a proxy like a register, routine documentation)</td><td>yes</td></tr>
<tr><td>I8</td><td>Was the same mode of data collection used for all patients?</td><td>yes</td></tr>
<tr><td>I9</td><td>Was the number of non-responders/ dropouts unlikely to affect the results?</td><td>yes</td></tr>
<tr><td colspan="3">Additional comments: Prospective data collection; data were collected between October 2005 and July 2006. GPs took a standardized history and performed a physical examination according to a CRF that was piloted and modified accordingly. They also recorded their preliminary diagnoses, investigations, and management related to the patients' chest pains. Patients were contacted by phone 6 weeks and 6 months after the index consultation. Study assistants blinded to clinical data already recorded asked about the course of patients' chest pain, treatments including hospitalization, and drugs. Discharge letters from specialists and hospitals were requested by GPs. Practices were visited at 4-week intervals to check CRFs, recruitment logs, and compliance with study procedures. Random audits were performed in order to search the routine documentation of participating practices to identify cases of chest pain not included in the study.
143 of 1355 chest pain patients did not participate or were excluded; reasons were provided.</td></tr>
<tr><td colspan="3"></td></tr>
<tr><td colspan="3">Domain C: Determination of the underlying etiology of the symptom</td></tr>
<tr><td colspan="2">Risk that the diagnostic work up introduce bias?</td><td>low</td></tr>
<tr><td colspan="3">Support for judgement:</td></tr>
<tr><td>I10</td><td>Was the etiologic category clearly defined?</td><td>no</td></tr>
<tr><td>I11</td><td>Was the diagnostic work up likely to correctly classify the respective etiology?</td><td>yes</td></tr>
<tr><td>I12</td><td>Did every patient receive the same diagnostic work up to detect the respective etiology?</td><td>no</td></tr>
<tr><td colspan="3">Additional comments: After 6 months, a reference panel consisting of one cardiologist, one GP, and one member of their search staff at the Department of Family Medicine reviewed the baseline and follow-up data of each patient. Analyzing all the information gathered during the follow-up period (results of further investigations, letters from specialists, hospital discharge reports, etc.), they decided on the most likely medical condition having caused an individual patient's chest pain at baseline.</td></tr>
<tr><td colspan="3"></td></tr>
</table>

Study ID: Verdon 2008 [25–29]

**Domain A: Selection of patients and GPs**		
Risk that the selection of patients introduced bias?		low
Concern that the selection of patients introduced substantial variation?		low
Support for judgement		
I1	Was the symptom to be investigated clearly described?	no
I2	Were the selection criteria of the patients clearly described?	yes
I3	Was a consecutive or random sample of patients enrolled?	yes
I4	Was it a multi-centered study?	yes
I5	Did the selection criteria of the patients permit the study population to represent the full spectrum of those presenting with the symptom in the respective setting?	yes
I6	Were the participating health care professionals/ institutions representative for the setting to be investigated in the review?	yes
Additional comments: Symptom: Chest pain was either already known or a new symptom, no further specific definition provided; the presence of chest pain was ascertained according to the usual practice of every GP in a pragmatic approach<br>patients and recruitment: GPs consecutively included every patient, aged over 16, presenting with chest pain as the main or an ancillary symptom were included. During a five-week period between March and June 2001. .<br>GPs: 58 GPs in western Switzerland and 5 residents of an academic primary care outpatient department. The practices were located both in urban and non-urban areas. However most of them were located relatively close to an emergency center. All participating primary care physicians were trained to handle, at least initially, emergency cases. Participating GPs had an average experience in private practices of 12 years (range 1 to 24).		
**Domain B: Data collection and patient flow**		
Risk that the mode of data collection and/ or patient flow introduced bias		low
Support for judgement		
I7	Were data about the symptom und the inclusion criteria collected directly from the patients (as opposed to a proxy like a register, routine documentation)	yes
I8	Was the same mode of data collection used for all patients?	yes
I9	Was the number of non-responders/ dropouts unlikely to affect the results?	yes
Additional comments: prospective data collection, no drop-out mentioned<br>An initial form was filled in to record general patient characteristics, the type, characteristics and location of chest pain, initial plausible etiologies, early diagnosis, detailed history and physical examination, level of anxiety expressed by patients and physicians, cardiovascular and thromboembolic risk factors, laboratory results made in emergency, comorbidities, medication and treatment decision at the end of the initial or index encounter. Decisions to refer the patient to an emergency center or to a specialist and to order tests were recorded. GPs decided the best possible work-up for their patient based on their own experience; The diagnosis retained at three and 12 months, possibly revised, further investigations treatments, hospitalizations and death were recorded. Follow-up questionnaires were filled in after three and twelve months and the patient was contacted. All completed forms were sent to the study coordination center. Researchers performed data entry checks, double data entry, and post entry checks. In addition, to ensure good data quality, before the launch of the study, participating GPs participated in a half-day training session to be introduced to the study and to learn how to fill in the questionnaires.		

**Domain C: Determination of the underlying etiology of the symptom**		
Risk that the diagnostic work up introduce bias?		low
Support for judgement:		
I10	Was the etiologic category clearly defined?	yes
I11	Was the diagnostic work up likely to correctly classify the respective etiology?	yes
I12	Did every patient receive the same diagnostic work up to detect the respective etiology?	no
Additional comments: All final one-year diagnoses were reviewed independently by a group of clinicians (FV, BF, LH, MJ) and discussed in case of incoherence. A precise final diagnosis was retained (for example metastasis or chest wall syndrome, and not only chest wall pain), derived from additional information collected during follow-up through case evolution, additional diagnostic or therapeutic testing, referral to specialists and hospitalization. The diagnoses retained after 12 months of follow-up were grouped in six clusters		

Study ID: Haasenritter 2012 [24]

**Domain A: Selection of patients and GPs**		
Risk that the selection of patients introduced bias?		low
Concern that the selection of patients introduced substantial variation?		unclear
Support for judgement		
I1	Was the symptom to be investigated clearly described?	yes
I2	Were the selection criteria of the patients clearly described?	yes
I3	Was a consecutive or random sample of patients enrolled?	yes
I4	Was it a multi-centered study?	yes
I5	Did the selection criteria of the patients permit the study population to represent the full spectrum of those presenting with the symptom in the respective setting?	unclear
I6	Were the participating health care professionals/ institutions representative for the setting to be investigated in the review?	yes
Additional comments: Symptom: pain, tightness, or oppression localized in the area between the clavicles and lower costal margins and anterior to the posterior axillary lines, acute or chronic<br>Patients and recruitment: Every patient above 35 years with a complaint of chest pain was to be included. Doctors were also asked to recruit at home visits and emergency calls. Patients were eligible irrespective of the acute or chronic nature of their complaint, or of previously known conditions including IHD or related risk factors. Patients whose chest pain had subsided for more than 1 month, whose chest pain had been investigated already, and/or who came for follow-up for chest pain were excluded.<br>GPs: 56 GPs in state of Hesse, Germany; details on GPs' characteristic (age, sex, experience in years, practice location) were provided.		
**Domain B: Data collection and patient flow**		
Risk that the mode of data collection and/ or patient flow introduced bias		low

Support for judgement		
I7	Were data about the symptom und the inclusion criteria collected directly from the patients (as opposed to a proxy like a register, routine documentation)	yes
I8	Was the same mode of data collection used for all patients?	yes
I9	Was the number of non-responders/ dropouts unlikely to affect the results?	yes
Additional comments: Data were collected between October 2005 and July 2006. GPs took a standardized history and performed a physical examination. They also recorded their preliminary diagnoses, investigations, and management related to the patients' chest pains. Patients were contacted by phone 6 weeks and 6 months after the index consultation. Study assistants blinded to clinical data already recorded asked about the course of patients' chest pain, treatments including hospitalization, and drugs. Discharge letters from specialists and hospitals were requested by GPs. 83 of 939 chest pain patients did not participate or were excluded; reasons were provided.		
**Domain C: Determination of the underlying etiology of the symptom**		
Risk that the diagnostic work up introduce bias?		low
Support for judgement:		
I10	Was the etiologic category clearly defined?	yes
I11	Was the diagnostic work up likely to correctly classify the respective etiology?	yes
I12	Did every patient receive the same diagnostic work up to detect the respective etiology?	no
Additional comments: After 6 months, a reference panel consisting of one cardiologist, one GP, and one member of their search staff at the Department of Family Medicine reviewed the baseline and follow-up data of each patient. Analyzing all the information gathered during the follow-up period (results of further investigations, letters from specialists, hospital discharge reports, etc.), they decided on the most likely medical condition having caused an individual patient's chest pain at baseline		

References

1. Rosser WW. An exploratory report of chest pain in primary care. A report from ASPN. *J Am Board Fam Pract* 1990; **3(3):** 143–50.

2. Sox HC, JR., Hickam DH, Marton KI, *et al.* Using the patient's history to estimate the probability of coronary artery disease: a comparison of primary care and referral practices. *Am J Med* 1990; **89(1):** 7–14.

3. Buntinx F, Truyen J, Embrechts P, *et al.* Chest pain: an evaluation of the initial diagnosis made by 25 Flemish general practitioners. *Fam Pract* 1991; **8(2):** 121–4.

4. Buntinx F, Truyen J, Embrechts P, *et al.* Evaluating patients with chest pain using classification and regression trees. *Fam Pract* 1992; **9(2):** 149–53.

5. Buntinx F, Knockaert D, Bruyninckx R, *et al.* Chest pain in general practice or in the hospital emergency department: is it the same? *Fam Pract* 2001; **18(6):** 586–9.

6. Klinkman MS, Stevens D, Gorenflo DW. Episodes of care for chest pain: a preliminary report from MIRNET. Michigan Research Network. *J Fam Pract* 1994; **38(4):** 345–52.

7. Svavarsdottir AE, Jonasson MR, Gudmundsson GH*, et al.* Chest pain in family practice. Diagnosis and long-term outcome in a community setting. *Can Fam Physician* 1996; **42:** 1122–8.

8. Katerndahl DA, Trammell C. Prevalence and recognition of panic states in STARNET patients presenting with chest pain. *J Fam Pract* 1997; **45(1):** 54–63.

9. Nilsson S, Ortoft K, Molstad S. The accuracy of general practitioners' clinical assessment of chest pain patients. *Eur J Gen Pract* 2008; **14(2):** 50–5.

10. Nilsson S, Scheike M, Engblom D*, et al.* Chest pain and ischaemic heart disease in primary care. *Br J Gen Pract* 2003; **53(490):** 378–82.

11. Scheike M, Nilsson S, Nylander E. Exercise testing and myocardial perfusion scintigraphy in primary care patients with chest pain of new onset. *Scand J Prim Health Care* 2007; **25(2):** 117–22.

12. Bruyninckx R, van den Bruel A, Aertgeerts B*, et al.* Why does the general practitioner refer patients with chest pain not-urgently to the specialist or urgently to the emergency department? Influence of the certainty of the initial diagnosis. *Acta Cardiol* 2009; **64(2):** 259–65.

13. Bruyninckx R, van den Bruel A, Aertgeerts B*, et al.* Half of the patients with chest pain that are urgently referred are transported in unsafe conditions. *Eur J Emerg Med* 2008; **15(6):** 330–3.

14. Bruyninckx R, van den Bruel A, Buntinx F*, et al.* Excess of mortality in patients with chest pain peaks in the first 3 days period after the incident and normalizes after 1 month. *Fam Pract* 2010.

15. Glombiewski JA, Donner-Banzhoff N, Bösner S*, et al.* The course of unspecific chest pain: Symptom persistence and inappropriate referrals. *European Journal of Pain* 2009; **13:** S30.

16. Bösner S, Becker A, Haasenritter J*, et al.* Chest pain in primary care: epidemiology and pre-work-up probabilities. *Eur J Gen Pract* 2009; **15(3):** 141–6.

17. Bösner S, Becker A, Abu Hani M*, et al.* Accuracy of symptoms and signs for coronary heart disease assessed in primary care. *Br J Gen Pract* 2010; **60(575):** 246–57.

18. Bösner S, Becker A, Hani MA*, et al.* Chest wall syndrome in primary care patients with chest pain: presentation, associated features and diagnosis. *Fam Pract* 2010; **27(4):** 363–9.

19. Bösner S, Haasenritter J, Hani MA*, et al.* Gender differences in presentation and diagnosis of chest pain in primary care. *BMC Fam Pract* 2009; **10:** 79.

20. Bösner S, Haasenritter J, Becker A*, et al.* Heartburn or angina? Differentiating gastrointestinal disease in primary care patients presenting with chest pain: a cross sectional diagnostic study. *Int Arch Med* 2009; **2:** 40.

21. Bösner S, Haasenritter J, Abu Hani M*, et al.* Accuracy of general practitioners' assessment of chest pain patients for coronary heart disease in primary care: cross-sectional study with follow-up. *Croat Med J* 2010; **51(3):** 243–9.

22. Bösner S, Haasenritter J, Becker A*, et al.* Ruling out coronary artery disease in primary care: development and validation of a simple prediction rule. *CMAJ* 2010; **182(12):** 1295–300.

23. Glombiewski JA, Rief W, Bösner S*, et al.* The course of nonspecific chest pain in primary care: symptom persistence and health care usage. *Arch Intern Med* 2010; **170(3):** 251–5.

24. Haasenritter J, Bösner S, Vaucher P*, et al.* Ruling out coronary heart disease in primary care: external validation of a clinical prediction rule. *The British journal of general practice : the journal of the Royal College of General Practitioners* 2012; **62(599):** e415-21.

25. Verdon F, Herzig L, Burnand B*, et al.* Chest pain in daily practice: occurrence, causes and management. *Swiss Med Wkly* 2008; **138(23-24):** 340–7.

26. Verdon F, Burnand B, Herzig L*, et al.* Chest wall syndrome among primary care patients: a cohort study. *BMC Fam Pract* 2007; **8:** 51.

27. Verdon F, Herzig L, Muehlemann N*, et al.* [The place of the clinic in primary case--the TOPIC study]. *Rev Med Suisse* 2009; **5(192):** 476–80.

28. Gencer B, Vaucher P, Herzig L*, et al.* Ruling out coronary heart disease in primary care patients with chest pain: a clinical prediction score. *BMC Med* 2010; **8:** 9.

29. Verdon F, Junod M, Herzig L*, et al.* Predictive ability of an early diagnostic guess in patients presenting with chest pain; a longitudinal descriptive study. *BMC Fam Pract* 2010; **11:** 14.
