# Supplementary material for: Causes of chest pain in primary care – a systematic review and meta-analysis
Source: Croat Med J. 2015 Oct;56(5):422–30. doi: 10.3325/cmj.2015.56.422 (PMC4655927; doi:10.3325/cmj.2015.56.422)
Supplement: Supplementary Material 1 [file CroatMedJ_56_s003.pdf]

Supplement 3: Forest plots

Etiology: Coronary heart disease (any)

Study	k	N	Percentage	95% CI
**Overall risk of bias = low**				
Buntinx 1991	31	318	9.7	[6.8; 13.7]
Nilsson 2008	61	516	11.8	[9.2; 15.0]
Verdon 2008	85	672	12.6	[10.3; 15.5]
Bösner 2009	179	1212	14.8	[12.8; 16.9]
Haasenritter 2012	93	856	10.9	[8.9; 13.2]
**Overall risk of bias = unclear or high**				
Rosser 1990	287	832	34.5	[31.3; 37.9]
Klinkman 1994	52	392	13.3	[10.1; 17.1]
Svavarsdottir 1996	34	190	17.9	[12.9; 24.3]
Katerndahl 1997	2	51	3.9	[0.7; 14.6]

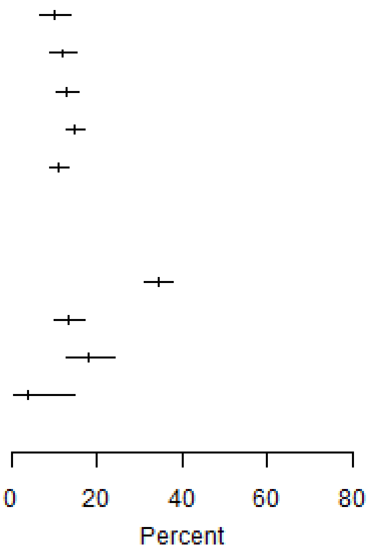

I²	Tau²	Prediction interval
96.6% (95.0-97.6%)	0.271	4.2-37.6%

k: number of patients with the respective etiology.
N: number of all patients presenting with chest pain.

Etiology: Coronary heart disease (stable)

Study	k	N	Percentage	95% CI
**Overall risk of bias = low**				
Sox 1990	23	289	8.0	[5.2; 11.9]
Buntinx 1991	21	318	6.6	[4.2; 10.1]
Verdon 2008	75	672	11.2	[8.9; 13.8]
Bösner 2009	135	1212	11.1	[9.5; 13.1]
Haasenritter 2012	71	856	8.3	[6.6; 10.4]
**Overall risk of bias = unclear or high**				
Klinkman 1994	41	392	10.5	[7.7; 14.0]

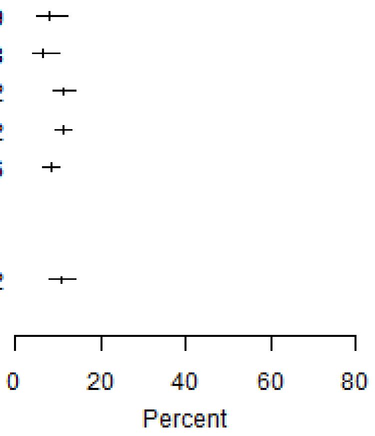

I²	Tau²	Prediction interval
54.2% (0.0-81.6%)	0.024	6.1-14.7%

k: number of patients with the respective etiology.
N: number of all patients presenting with chest pain.

Etiology: Acute coronary syndrome/ myocardial infarction

Study	k	N	Percentage	95% CI
**Overall risk of bias = low**				
Buntinx 1991	10	318	3.1	[1.6; 5.9]
Verdon 2008	10	672	1.5	[0.8; 2.8]
Bösner 2009	44	1212	3.6	[2.7; 4.9]
Haasenritter 2012	22	856	2.6	[1.7; 3.9]
**Overall risk of bias = unclear or high**				
Rosser 1990	24	832	2.9	[1.9; 4.3]
Klinkman 1994	6	392	1.5	[0.6; 3.5]
Svavarsdottir 1996	4	190	2.1	[0.7; 5.7]

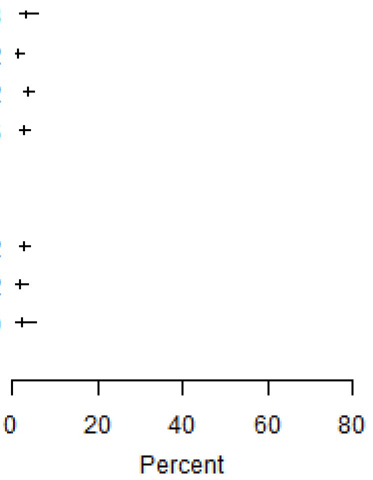

I²	Tau²	Prediction interval
40.4% (0.0-74.9%)	0.048	1.4-4.9%

k: number of patients with the respective etiology.
N: number of all patients presenting with chest pain.

Etiology: Cardiovascular diseases

Study	k	N	Percentage	95% CI
**Overall risk of bias = low**				
Buntinx 1991	44	318	13.8	[10.3; 18.2]
Verdon 2008	108	672	16.1	[13.4; 19.1]
**Overall risk of bias = unclear or high**				
Bryninckx 2009	741	1996	37.1	[35.0; 39.3]

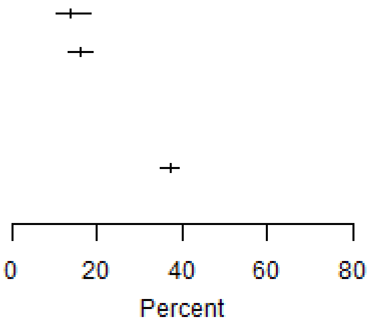

I²	Tau²	Prediction interval
98.6% (97.5-99.2%)	0.494	0.0-100.0%

k: number of patients with the respective etiology.
N: number of all patients presenting with chest pain.

Etiology: Gastrointestinal disorders

Study	k	N	Percentage	95% CI
**Overall risk of bias = low**				
Buntinx 1991	31	318	9.7	[6.8; 13.7]
Verdon 2008	55	672	8.2	[6.3; 10.6]
Bösner 2009	68	1212	5.6	[4.4; 7.1]
**Overall risk of bias = unclear or high**				
Rosser 1990	114	832	13.7	[11.5; 16.3]
Klinkman 1994	74	392	18.9	[15.2; 23.2]
Svavarsdottir 1996	7	190	3.7	[1.6; 7.7]
Katerndahl 1997	5	51	9.8	[3.7; 22.2]
Bryninckx 2009	161	1996	8.1	[6.9; 9.4]

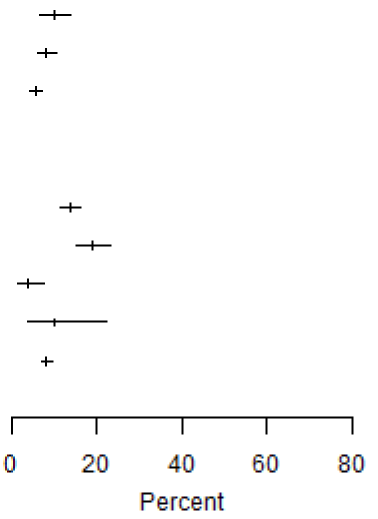

I²	Tau²	Prediction interval
92.1% (86.8-95.3%)	0.247	2.7-27.0%

k: number of patients with the respective etiology.
N: number of all patients presenting with chest pain.

I²	Ae	
93.9% (85.5-97.4%)	0.377	0.0-99.9%
k: number of patients with the respective etiology.<br>N: number of all patients presenting with chest pain.		

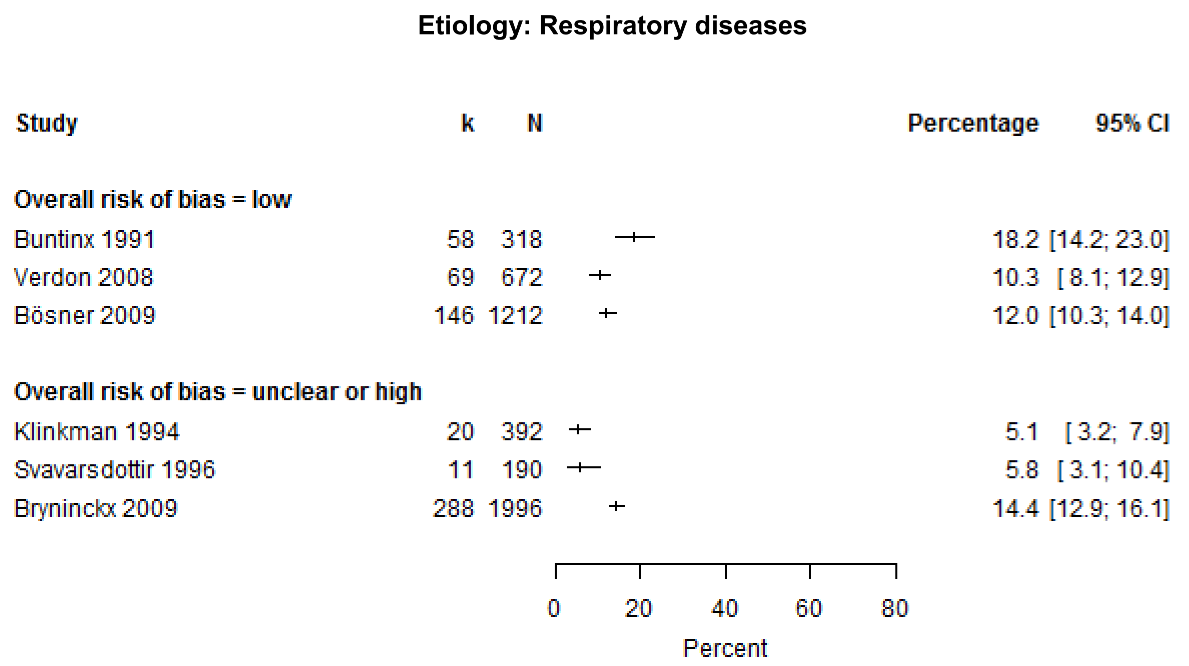

I² (95% CI)	Tau²	Prediction interval
88.8% (78.1-94.2%)	0.256	2.5-35.0%
k: number of patients with the respective etiology.<br>N: number of all patients presenting with chest pain.		

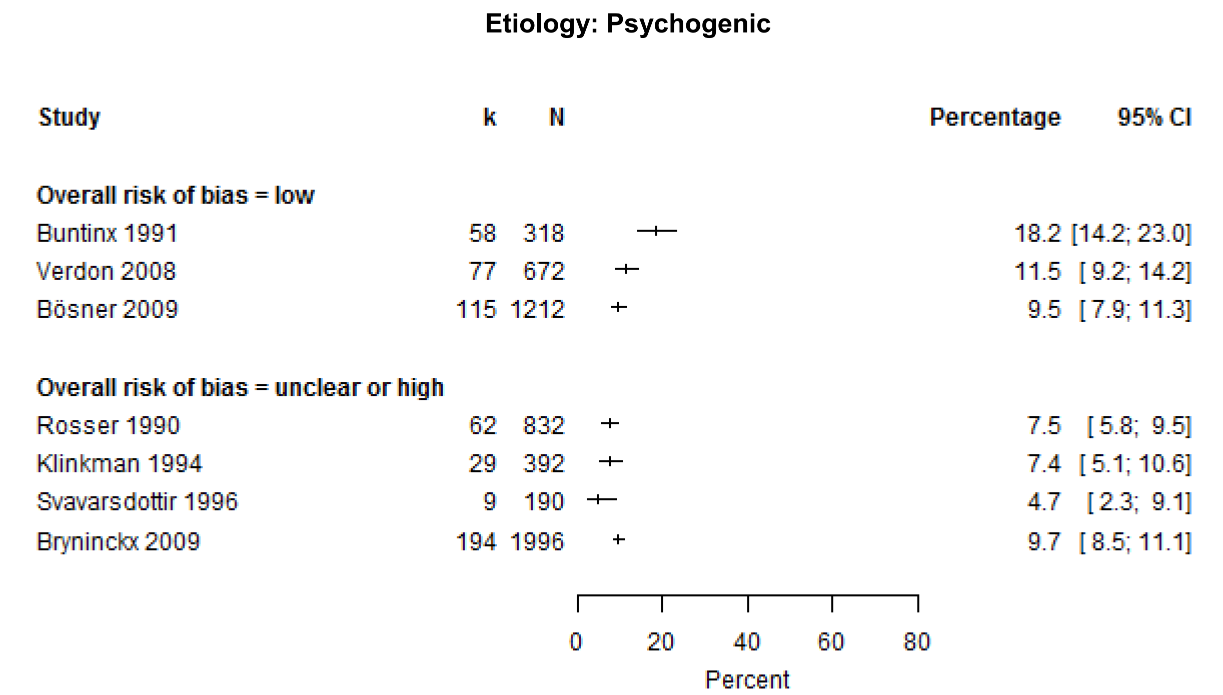

I²	Tau²	Prediction interval
84.8% (70.6-92.2%)	0.148	3.5-23.4%

k: number of patients with the respective etiology.
N: number of all patients presenting with chest pain.

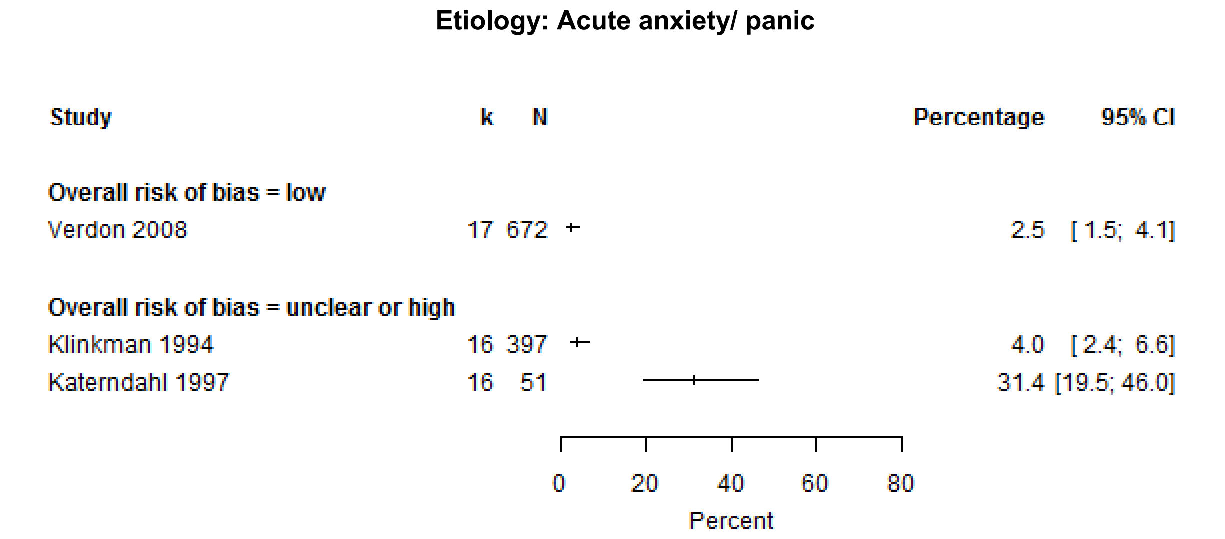

I²	Tau²	Prediction interval
96.6% (92.9-98.4%)	2.271	0.0-100.0%

k: number of patients with the respective etiology.
N: number of all patients presenting with chest pain.

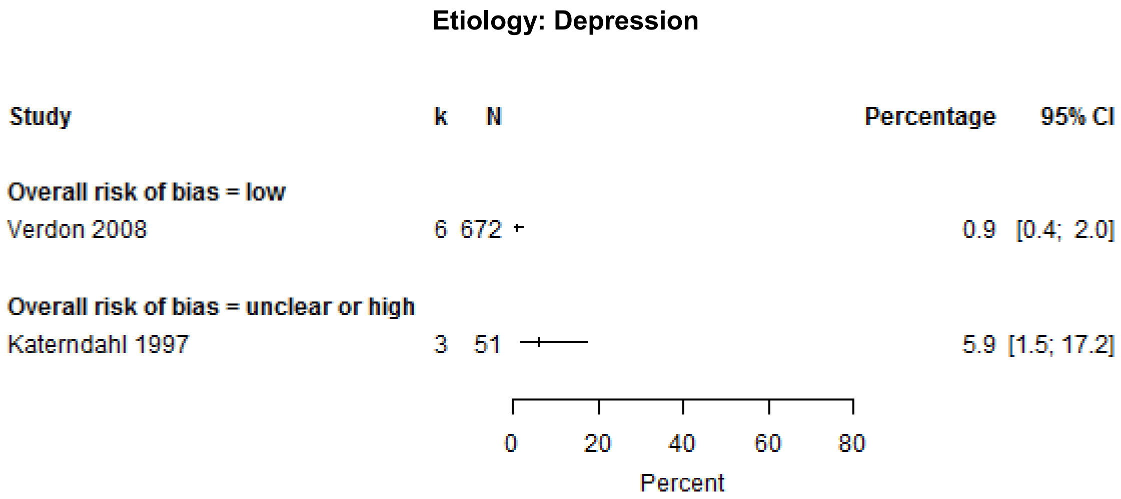

I^2	Tau^2	Prediction interval
86.1% (NA)	1.615	NA

k: number of patients with the respective etiology.
N: number of all patients presenting with chest pain.

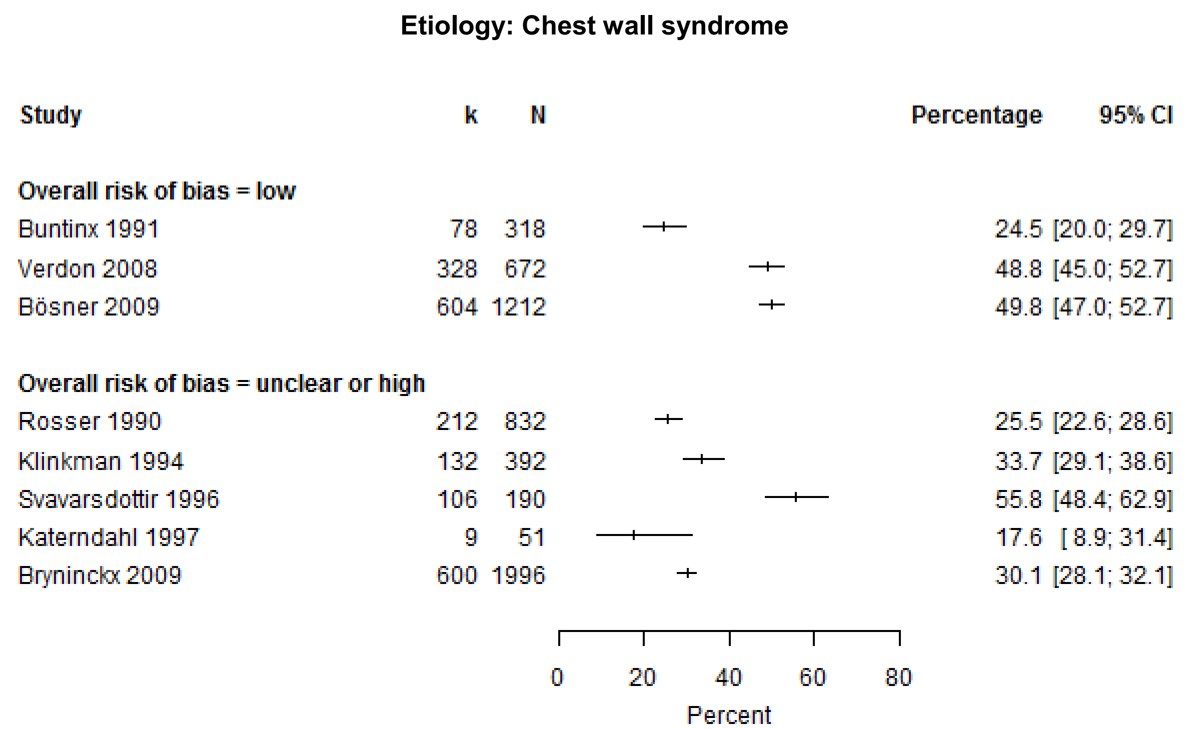

I²	Tau²	Prediction interval
97.4% (96.2-98.2%)	0.34	10.7-71.4%

k: number of patients with the respective etiology.
N: number of all patients presenting with chest pain.

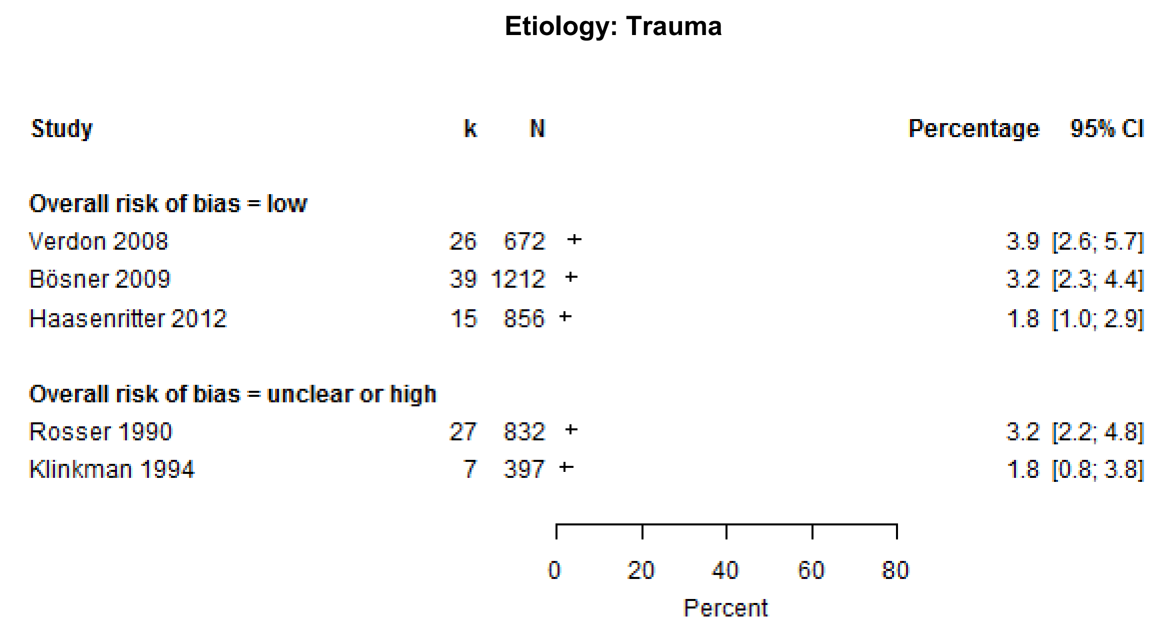

I^2	Tau^2	Prediction interval
53.5% (0.0-82.9%)	0.061	1.1-6.8%

k: number of patients with the respective etiology.
N: number of all patients presenting with chest pain.
